# The Biochar Amendment Improves Tomato Growth and Yield under Deficit Irrigation in Sandy Loam Soil in Senegal

**DOI:** 10.1155/2024/9945354

**Published:** 2024-07-11

**Authors:** Simeon Diedhiou, Alassane Maiga, Philippe B. Himbane, Maduabuchi P. Iboko, Lat Grand Ndiaye, Ibrahima Diedhiou

**Affiliations:** ^1^ University of Sciences, Techniques and Technologies of Bamako (West African Science Service Centre on Climate Change and Adapted Land Use) Rural Polytechnic Institute for Training and Applied Research (IPR/IFRA), Bamako, Mali; ^2^ Institute of Rural Economy (IER), Bamako, Mali; ^3^ University Assane Seck of Ziguinchor Department of Physics, Ziguinchor, Senegal; ^4^ University of Thiès National School of Agriculture (ENSA), Thiès, Senegal

## Abstract

Poor agricultural soil management practices and water use optimisation in irrigation are major challenges facing crop production in Senegal. To address these problems, a factorial experiment was conducted in 2021 and 2022 to investigate the effects of biochar on tomato growth and yield in sandy loam soil under different irrigation levels. Treatments included three biochar treatments (B2 = 30 t·ha^−1^, B1 = 15 t·ha^−1^, and B0 = 0 t·ha^−1^) and three irrigation levels (full irrigation, W0 = 8 L·m^−2^·day^−1^; medium deficit irrigation, W1 = 6 L·m^−2^·day^−1,^ which is 75% of W0; and deficit irrigation, W2 = 4 L·m^−2^·day^−1^, 50% of W0). The results showed that using biochar at 30 t·ha^−1^ significantly (*P* < 0.05) reduced the bulk density of the soil by up to 8.3% under W1. In addition, biochar at 15 t·ha^−1^ and 30 t·ha^−1^ enhanced, regardless of the amount of water applied, the growth of tomato plants by at least 14% compared to that in the B0 treatment. Furthermore, the tomatoes' yields in biochar treatments B1 (12.58 t·ha^−1^) and B2 (12.45 t·ha^−1^) under W2 were greater than those under B0 (9.27 t·ha^−1^) under full irrigation. The combinations of biochar and the lowest irrigation water level (W2 and B1 or W2 and B2) can therefore allow a water economy of up to 50% of full irrigation without compromising yield. Our study concluded that biochar could sustainably reduce agricultural water consumption while increasing yields. To further understand the influence of biochar on sandy loam soil, more research is needed on its effects on soil moisture content at permanent wilting points and field capacity.

## 1. Introduction

Water is one of the resources that is needed in many industrial activities [1, 2]. Following the increase in the world population since the 1950s, freshwater demand is growing in different sectors [3]. Agriculture uses 72% of that freshwater, and in some developing countries, it is up to more than 90% [4]. The gap in water distribution among nations is exacerbated by climate change, with less available water for irrigation, mainly in the arid regions that will experience more drought. Climate change will affect water availability and use through variations in rainfall patterns and water stocks [5]. With the growth of the continent's population and climate change, this quantity of fresh water will surely decrease [6]. Thus, access to water will be more challenging [7] for all activities.

In Senegal, the long dry season, the decrease in rainfall amount, water pollution, and the increase in population led to a decrease in freshwater resources and put it under pressure and competition among the users [8]. Inadequate agricultural water management combined with the weak capacity of most Senegalese soils to hold water will lead to more inefficient irrigation water use.

Tomato is one of the main horticultural crops produced in large quantities both in the open field and under greenhouse production in many countries [9, 10]. Nicolas et al. [11] stated that tomato is the second most consumed vegetable in the world after potato. In Senegal, tomato is the second most important horticultural crop after onion, with approximately 20% of the total horticultural area [12]. Most of the Senegalese tomato production is produced under irrigation. Thus, associated with the requirement for plant water, bad agricultural water management practices, and climate change-related issues, it represents a great source of water consumption and competition for other human water uses [13, 14].

Biochar has been used to improve irrigation water management and increase the water retention capacity of the soil [15]. Werdin et al. [16] found that low-density eucalyptus feedstock has a 35% higher water holding capacity and 45% more water available to plants than biochar made from high-density eucalyptus. According to Gray et al. [17], biochar increases the soil water holding capacity through its high porosity and hydrophilic oxygen-containing functional groups. Mao et al. [18] stated that a decrease in hydrophobicity increases nutrient retention, soil particle aggregation, and water retention. Under wet conditions, biochar application is estimated to induce a 3–226% increase in wet aggregate stability [19] and a 4–130% increase in soil water depending on the rate of biochar application and soil properties [19, 20]. Liu et al. [21] found that biochar combined with half root zone irrigation at 70% field capacity improved tobacco yield. Faloye et al. [22] confirmed that biochar-deficit irrigation improved maize yield.

Regarding tomatoes, field works on the combination of biochar-deficit irrigation are rare. Although such kind of work on tomato exists, an increase of more than 80% in tomato yield has been found in biochar-treated plots compared with non-biochar-treated plots under deficit irrigation [23]. However, most studies on the application of biochar to improve soil water holding capacity for tomato production were conducted in the temperate zone, either in a lab or in a greenhouse [13, 14, 21, 23, 24].

In West Africa, researchers are trying to better understand the relationship between biochar, soil fertility status, and water holding capacity. Dugan et al. [25] evaluated the effect of biochar from sawdust, maize stover, and charcoal on three soil types in Ghana. In Senegal, Faye et al. [26] assessed the long-term effect of biochar on increasing fertilizer efficiency, CO_2_ sequestration, and pH in sandy soil.

Despite promising results, limited research has been conducted on the effects of biochar on soil water use efficiency and tomato yields in Senegal. Therefore, this study aimed to assess the efficiency of biochar in combination with deficit irrigation to improve tomato growth and yield in sandy loam soil in Senegal.

## 2. Materials and Methods

### 2.1. Study Area

The study was carried out under field conditions at the Ecole Nationale Supérieure d'Agriculture (ENSA) at the Agricultural Technology Application Centre (ATAC/CATA in French) (14°45′50″N; 16°53′24″W). The area has a semiarid climate, with an average annual precipitation and temperature of 500 mm and 27°C, respectively [27, 28]. The air temperature varies greatly throughout the day and year. The rainy season lasts from July to October and is typically characterized by scattered, high-intensity, episodic drought, and short-duration showers [29]. The dry season lasts from November to June and is divided into a cool dry season (November to February) and a hot dry season (March to June) [30, 31]. The relative humidity of the study region was 75% on average. The general properties of the soil at the start of the experiment are shown in Table 1.

### 2.2. Biochar Production

The biochar applied in this study was produced with local technology at the University of Assane Seck of Ziguinchor in the southern Senegal region. The technology consisted of a 200 L open-head steel barrel and a lid. The barrel measured 57.15 cm in diameter and 83.82 cm in height. The lid was topped by a 10 cm diameter chimney. The bottom of the barrel was perforated with small nails to allow minimal air exchange. During the production process, the empty barrel was mounted on stones (15 to 20 cm high). The barrel-stone assembly was covered with wet sand to limit free air circulation. Four holes were made in the wet sand to allow intermittent air movement between the openings at the base of the barrel and the chimney. The barrel was gently filled with peanut shells and lit from the top with dry grass. Subsequently, the lid with the chimney was closed to allow anaerobic combustion. The pyrolysis temperature was monitored every 10 minutes with a probe linked to a thermometer capable of reaching 1000°C, which was inserted through a small hole in the barrel. The average pyrolysis temperature was 800°C, and the residence times for pyrolysis were 1 hour and 30 minutes, followed by 3 hours of cooling in hermetically sealed barrels. The color of the smoke from the chimney was used to monitor the pyrolysis. Typically, the smoke color progressed from a brownish color, gradually turned blue, and became less dense at the end of the process. The obtained biochar was grounded, and three subsamples were taken to the University Assane Seck of Ziguinchor Laboratory to determine the chemical and physical properties of the biochar (Table 1).

### 2.3. Experimental Design and Field Management

The study was carried out from February to May 2021 and 2022 and comprised factorial combinations of biochar and water at three levels each. The combinations resulted in a total of 9 treatments (Table 2) and were as follows: biochar at 0 t·ha^−1^ (B0 = control), 15 t·ha^−1^ (B1), and 30 t·ha^−1^ (B2), while for the water level, we have W0, which corresponds to full irrigation (8 L·m^−2^·day^−1^ irrigation); W1 (6 L·m^−2^·day^−1^ irrigation), which is 75% of W0; and W2 (4 L·m^−2^·day^−1^ irrigation), which is 50% of W0. Treatments were arranged in a split plot design and replicated three times. The sizes of the main plot were 7.5 m × 6 m (45 m^2^), and the plots were separated by 5 m of buffer. The subplots were 2.5 m × 2 m (5 m^2^) each and were separated by a 1-m space between plots. The sieved biochar was evenly spread on the soil surface on January 22, 2021, and thoroughly incorporated into the 0–20-cm soil layer using a hand hoe. Biochar was applied once at the beginning of the experimental year 2021, and no additional biochar was applied during the experiment.

On 1 February and 5 February 2021 and 2022, representing the beginning of the 2021 and 2022 experimental years, respectively, *Solanum lycopersicum* L. var. MONA F1 tomato plants used for the experiment were established and the seedlings were subsequently transplanted (0.5 × 0.5) into the field after 21 days. One week before transplantation, 50% of the irrigation water needed for each treatment was applied. After that, each treatment was watered every morning between 7 am and 9 am, according to its water treatment. W0 = 8 L·m^−2^·day^−1^ (full irrigation); W1 = 6 L·m^−2^·day^−1^; W2 = 4 L·m^−2^·day^−1^. The same water management practices were used for both growing years. Similarly, for each year, 400 kg·ha^−1^ NPK fertilizer (10 : 10 : 20) was applied to all plots 10 days after transplantation. The applications included three splits: 50% basal, 20% first topping, and 30% second top dressing.

### 2.4. Data Collection

During the experiment and for each year, plant height and root biomass data, as well as tomato yield, were collected. The height of the plant was measured 30 (vegetative), 60 (flowering), and 80 (fruit formation stage) days after transplantation (DAT). Regarding the sampling method for plant height, six plants in the centre of each subplot were tagged. For root biomass, we used all plants from each subplot. The soils at the base of the plants were first thoroughly wetted, and approximately 5 cm from the plant, a hole was dug around the plant to allow lifting of the whole mass of the soil together with the plant and its roots. The sample was then taken as a whole and dipped into a bucket of water. The soil was allowed to freely recover from the plant. Subsequently, the whole plant and roots were removed. The root was cut from the base of the stalk, dried in the sun, and weighed.

At the end of the experiment, soil samples were also collected from each of the 27 subplots and transported to the laboratory to determine the bulk density of the soil. In addition, the tomato fruits were harvested, and the yield was determined for all the tomato fruits in each plot.

### 2.5. Water Use Efficiency

The efficiency of water use was determined by dividing the grain yield by the total amount of irrigation water applied throughout the whole season of production [35].

### 2.6. Statistical Analysis

Statistical analyses were performed using R Studio version 4.2.0 (citation). Two-way split plot ANOVA was used to analyse the effects of treatment on tomato growth, yield, and soil parameters. A significant difference was established at *P* < 0.05, where *P* is the significant mean separation analysis performed using the Duncan multiple range test.

## 3. Results

### 3.1. Soil Bulk Density

**Figure 1:**
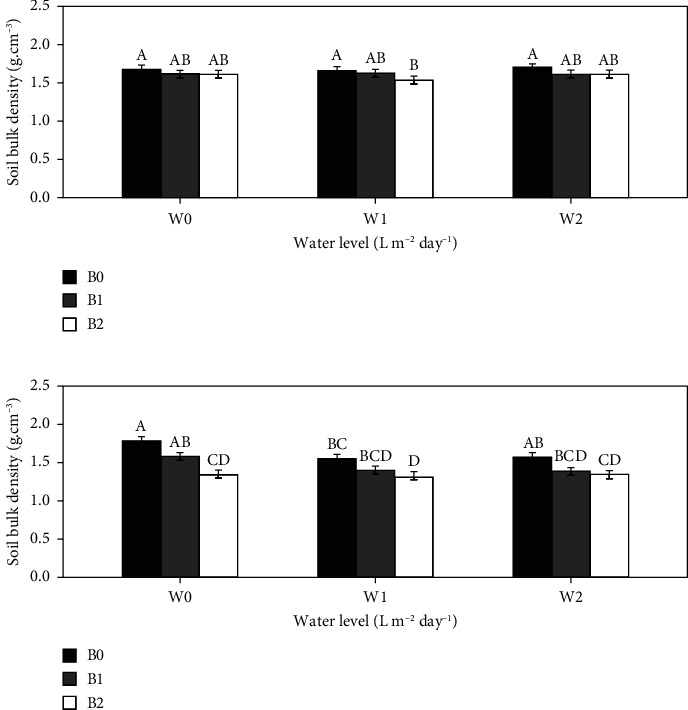
Effect of biochar on soil bulk density for the 2021 (a) and 2022 (b) growing seasons. Bars with the same uppercase letter are not significantly different at *P* < 0.05, where W0 = 8 L·m^−2^·day^−1^ (full irrigation); W1 = 6 L·m^−2^·day^−1^; W2 = 4 L·m^−2^·day^−1^ and B0 (control) = 0 t·ha^−1^; B1 = 15 t·ha^−1^; B2 = 30 t·ha^−1^.

Regardless of the irrigation regime, the results showed that biochar treatments led to a lower bulk density than nonbiochar treatments. The highest and lowest bulk density values were obtained with the combination of full irrigation (W0 = 8 L·m^−2^·day^−1^) and B0 = 0 t·ha^−1^ of biochar (W0B0 = 1.79) and the combination of 75% of full irrigation (W1 = 6 L·m^−2^·day^−1^) and B2 = 30 t·ha^−1^ of biochar (W1B2 = 1.33), respectively. However, in 2021 (Figure 1(a)), the differences were significant only under the deficit irrigation W1 (*P* < 0.05), with W1B2 showing a bulk density up to 8.3% lower than that of W1B0. In 2022 (Figure 1(b)), under all irrigation regimes, only B2 showed a significant difference compared to B0 (*P* < 0.05). The bulk density of B2 was lower than that of B0 by up to 24.6%, 14.7%, and 14.5% under W0, W1, and W2 (50% of full irrigation = 4 L·m^−2^·day^−1^), respectively. There was no significant difference in the interaction effect between water level and biochar amount (*P* > 0.05).

### 3.2. Plant Height

**Figure 2:**
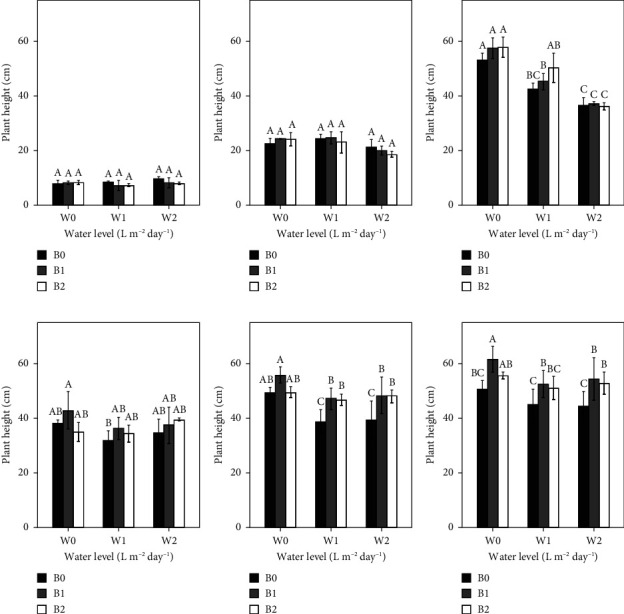
Effect of biochar at 30 DAT (a), 60 DAT (b), and 80 DAT (c) in the 2021 growing season and at 30 DAT (d), 60 DAT (e), and 80 DAT (f) in the 2022 growing season on plant height. Bars with the same uppercase letter are not significantly different at *P* < 0.05, where W0 = 8 L·m^−2^·day^−1^ (full irrigation); W1 = 6 L·m^−2^·day^−1^; W2 = 4 L·m^−2^·day^−1^ and B0 (control) = 0 t·ha^−1^; B1 = 15 t·ha^−1^; B2 = 30 t·ha^−1^.

The interaction between the irrigation regime and the applied biochar amount did not have a significant effect on plant height in either year (*P* > 0.05). In 2021 (Figures 2(a), 2(b), and 2(c)), the irrigation regime significantly (*P* < 0.05) affected tomato growth, but only at 80 days after transplantation (DAT), W0 showed the tallest plants. On the contrary, in 2022 (Figures 2(d), 2(e), and 2(f)), only biochar impacted tomato growth, and this effect began at 60 DAT (Figure 2(e)). Specifically, all biochar treatments under reduced irrigation regimes significantly increased tomato growth (*P* < 0.05) compared with B0. Relative to B0 treatment, height differences in B1 (15 t·ha^−1^ of biochar) and B2 treatments under W1 were 17.5% and 16.6%, respectively (Figure 2(e)). In addition, under W2, the differences were slightly greater, with B1 plants being 18.4% higher than those of B0, and B2 plants 18.25% higher than those of B0. At 80 DAT (Figure 2(f)), all B1 treatments significantly increased tomato height by 18%, 14.06%, and 18.16% under W0, W1, and W2, respectively, compared to B0. On the other hand, the increase in tomato growth in the B2 treatment was significantly greater than that in the B0 treatment only under W2.

### 3.3. Root Biomass

**Figure 3:**
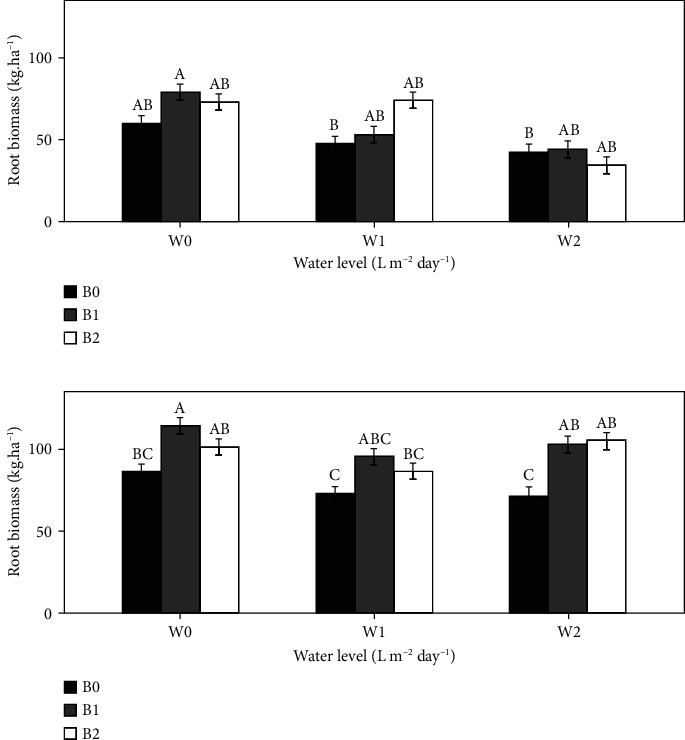
Effect of biochar on the development of tomato root biomass in the 2021 (a) and 2022 (b) growing seasons. Bars with the same uppercase letter are not significantly different at *P* < 0.05., where W0 = 8 L·m^−2^·day^−1^ (full irrigation); W1 = 6 L·m^−2^·day^−1^; W2 = 4 L·m^−2^·day^−1^ and B0 (control) = 0 t·ha^−1^; B1 = 15 t·ha^−1^; B2 = 30 t·ha^−1^.

During the 2021 growing season (Figure 3(a)), the different treatments and their combination did not have a significant effect on the root biomass (*P* > 0.05). However, in 2022 (Figure 3(b)), in all irrigation regimes, the root biomass of the tomato plants in treatment B1 was significantly different (*P* < 0.05) from that in treatment B0. Under the W0, W1, and W2 irrigation regimes, treatment B1 showed an increase in root biomass of 24.3%, 28.1%, and 34.71%, respectively, compared to B0. However, for root biomass in B2 treatments, the increases were 21.1% and 35.3% under W1 and W2, respectively, compared to B0 (*P* < 0.05).

### 3.4. Fruit Yield

**Figure 4:**
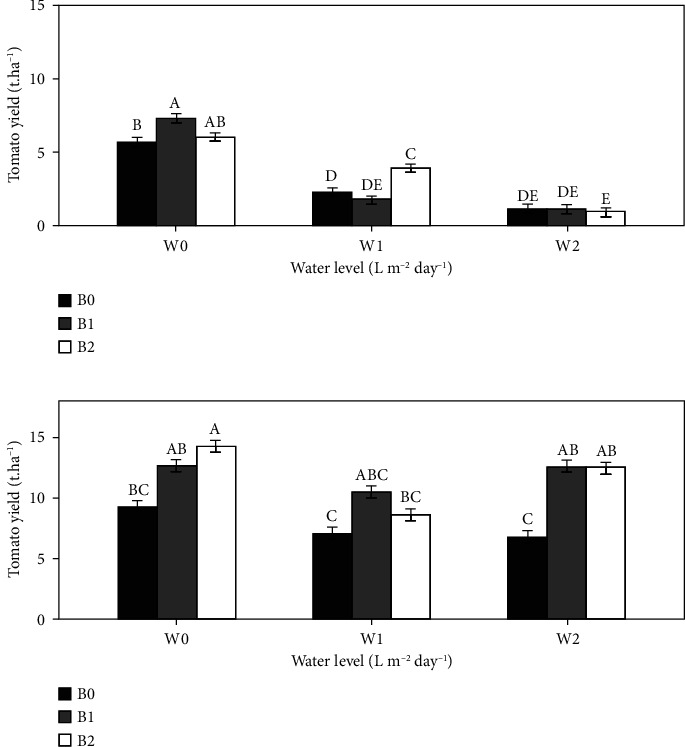
Effect of biochar on tomato yield in the 2021 (a) and 2022 (b) growing seasons. Bars with the same uppercase letter are not significantly different at *P* < 0.05, where W0 = 8 L·m^−2^ day^−1^ (full irrigation); W1 = 6 L·m^−2^·day^−1^; W2 = 4 L·m^−2^·day^−1^ and B0 (control) = 0 t·ha^−1^; B1 = 15 t·ha^−1^; B2 = 30 t·ha^−1^.

The results for 2021 (Figure 4(a)) revealed a significant interaction effect (*P* < 0.05) between the irrigation dose and the amount of biochar applied. Compared to W0B0, the yield of W0B1 increased by 21.8%. Under reduced irrigation, W1B2 increased the yield by 42.09% compared with W1B0, and no significant differences were detected between W2B1, W2B2, and W2B0. In 2022 (Figure 4(b)), the interaction effect between water level and biochar amount was no longer significant (*P* > 0.05). The different biochar treatments (B1 and B2) resulted in higher tomato yields than the nonbiochar treatment (B0) in all irrigation regimes (W0, W1, and W2). Furthermore, B1 (12.58 t·ha^−1^) and B2 (12.45 t·ha^−1^) under W2 resulted in greater tomato yields than B0 (9.27 t·ha^−1^) under full irrigation (W0). However, the differences were significant (*P* < 0.05) only in the irrigation regime W0, with B2 showing a 34.7% higher yield than B0, and under W2, with B1 and B2 showing yields greater than B0 by 46.1% and 45.7%, respectively.

### 3.5. Relation between Root Biomass and Yield

**Figure 5:**
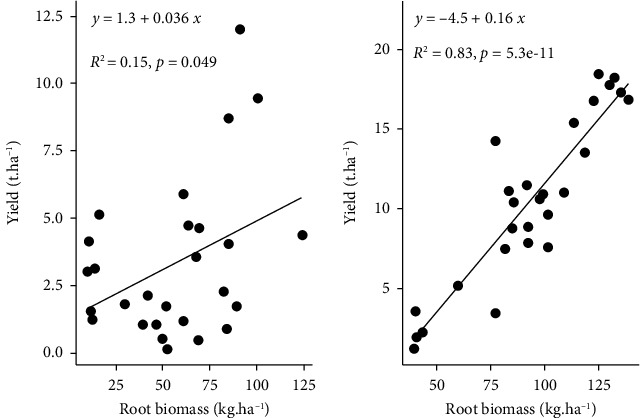
Correlations between tomato yield and root biomass in the 2021 (a) and 2022 (b) growing seasons.

In the 2021 and 2022 experiments, there was a significant (*P* < 0.05) positive correlation between root biomass and yield. However, in 2021 (Figure 5(a)), the correlation between root biomass and yield was weak (15%). This correlation was stronger in 2022 (Figure 5(b)), with 83% between root biomass and tomato yield.

### 3.6. Water Use Efficiency

**Figure 6:**
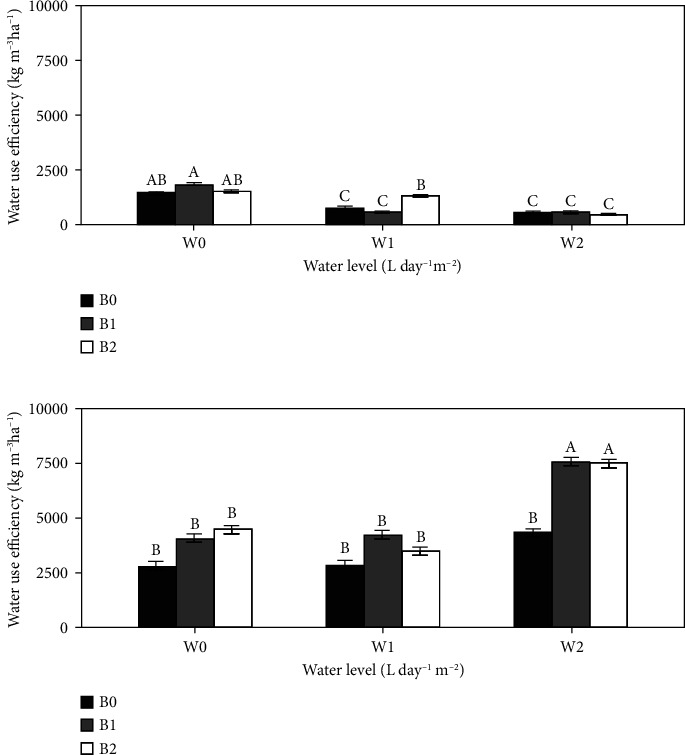
Effect of biochar on water use efficiency in the 2021 (a) and 2022 (b) growing seasons. Bars with the same uppercase letter are not significantly different at *P* < 0.05, where W0 = 8 L·m^−2^·day^−1^ (full irrigation); W1 = 6 L·m^−2^·day^−1^; W2 = 4 L·m^−2^·day^−1^ and B0 (control) = 0 t·ha^−1^; B1 = 15 t·ha^−1^; B2 = 30 t·ha^−1^.

During the 2021 experiment (Figure 6(a)), only B2 under W1 resulted in 42.2% lower water consumption (*P* < 0.05) to produce tomato than B0. In 2022 (Figure 6(b)), B1 and B2 had significantly (*P* < 0.05) greater water use efficiency than B0 by 46% and 45.5%, respectively, only under W2. The interaction effect between water level and biochar was not significant (*P* > 0.05) during the two growing seasons.

## 4. Discussion

### 4.1. Bulk Density and Tomato Development

Our findings indicate that the application of biochar improved the bulk density of sandy loam soil, particularly during 2022 growing season. Similar results have been observed by Ghorbani et al. [36] in loamy sand and clay soils. This effect could be due to the composition of the biochar itself, which has a bulk density typically lower than that of the soil in which it is incorporated into, as Blanco-Canqui [37] suggested. Furthermore, our study revealed that the amount of biochar applied can also impact soil bulk density. Generally, B2 decreased more the bulk density compared to B1, especially in 2022 growing season. These results align with the findings of Yu et al. [38], who noted that the soil bulk density decreases with increasing amounts of biochar up to a threshold of 60 t·ha^−1^, after which no effect is observed. When the bulk density of the soil decreases, the porosity of the soil increases directly. Our locally produced biochar is a mixture of different particle sizes (Table 1) which positively affects porosity at all levels, including micropores, mesopores, and macropores. The variety of biochar particle sizes is beneficial for improving soil porosity, as discovered by Alghamdi et al. [39], who identified a correlation between the size of biochar particle size and its impact on soil pores. Liu et al. [40] also reported that each pore size specifically affects soil water retention. Therefore, the mixture of biochar particles contributes to the retention of water and movement into the soil, such as infiltration. The influence of biochar particle size on porosity, which is closely related to bulk density, can increase soil moisture content at field capacity. This increase in soil field capacity is an excellent indicator of enhanced microporosity. The reduction in the bulk density of the soil, which can affect the retention of soil water, showed an immediate improvement. However, the positive impact of biochar on plant growth was only noticeable during 2022 growing season. Faye et al. [26] suggested that the benefits of biochar in soil and crops likely increase with time. This long-term effect may explain the distinctive effect of biochar observed in the second season, indicating its long-term potential.

Regardless of the amount, the application of biochar enhanced root biomass (Figure 3(b)). The increase in plant roots under biochar application may be related to an increase in soil nutrients due to the application of biochar. Furthermore, biochar increases soil aeration, which can also increase root respiration and growth [41], guaranteeing greater access to water and nutrients [42]. Our findings contrast with those of Busscher et al. [43], who reported that biochar application cannot reduce penetration resistance at rates lower than 44 t·ha^−1^. Root development in soil could be related to the type of soil, which could lead to differences in penetration resistance, and the duration of biochar into the soil, which was relatively long in this study (two years) compared to most studies (a few months) related to penetration resistance [43, 44]. Thus, the good rooting system under W2B1 and W2B2 could explain the difference in tomato growth between the two seasons. In addition, a decrease in bulk density could result in an increase in soil biota, which could make the soil more porous [45]. Lehmann et al. [46] confirmed through a review paper that biochar can effectively increase the abundance of soil microorganisms, which is a very important parameter for plant growth and development. Zwieten et al. [47] reported that improvements in the activities of certain microorganisms are responsible for the positive changes that occur after biochar application.

### 4.2. Tomato Yield and Water Use Efficiency

The results of the 2021 and 2022 experiments indicated that the influence of biochar on the yield of tomatoes can be divided into two phases: the combined water and biochar regulation phase, which occurs in the 2021 growing season, and the nutrient and biochar effect phase, which occurs in the 2022 growing season (Figure 4(b)). This trend in the yield of the first season could be explained by the effect of the size of the biochar particles on the soil aggregates and, by extension, soil moisture. According to Aslam et al. [48], biochar in soil can generate intrapores based on its particle size. Smaller particles (<2 mm) affect micro- and mesoporosity, thus increasing soil water retention capacity. At the same time, larger particles create new pores, probably larger than soil's macropores, which could regulate water fluxes. In 2022, the effects of biochar treatments on tomato yield were more pronounced than those of the control, regardless of the irrigation regime. The correlation between root development and tomato yield increased from the first year (Figure 5(a)) to the second year (Figure 5(b)), increasing from 15% to 83%. This greater effect of biochar in 2022 might be due to the residual influence of biochar, as it is recalcitrant. Akhtar et al. [49] reported similar results; they obtained higher tomato yields in response to reduced irrigation applied in biochar treatments than in full irrigation without biochar. In addition, these results could be linked to the gradual increase in the effect of biochar on soil water retention and nutrient status over the years of production. The results revealed that to enhance the effectiveness of biochar for relatively short-term use and meet crop water and nutrient needs of at least 50%, biochar could be activated or incubated for at least one year before use, as suggested by Esmaeelnejad et al. [50]. The authors found in their biochar incubation experiment that total porosity, particularly mesoporosity, increased over incubation days, with an increase of 23.8% between 0 and 180 days in certain cases.

Regarding the water use efficiency, the results showed that it was optimal with biochar amendment combined with 50% of ordinary irrigation as practices in tomato production in Senegal. Similar results were reported by Agbna et al. [13] and Ebrahimi et al. [15], who confirmed that a combination of deficit irrigation and biochar could increase the efficiency of irrigation water use. However, Aller et al. [51] reported no effect of biochar on water use in sandy loam soil after biochar application. According to Amoakwah et al. [52], biochar application rate below 10 t/ha did not have any significant impact on the main driver of water use efficiency in sandy loam soil. Our results suggest that the combination of 30 t·ha^−1^ of biochar with 4 L·m^−2^·day^−1^ of irrigation water in sandy loam soil could reduce the irrigation water requirement by 50% without compromising yields. These findings correlate perfectly with the results obtained from Akhtar et al. [49].

## 5. Conclusions

Due to its increasing capacity to ensure greater water management in the soil, by altering physical properties such as soil bulk density and porosity, biochar at 15 t·ha^−1^ and 30 t·ha^−1^ can lead to optimum production with a half reduction in the ordinary water supply in sandy loam soil in Senegal. The application of biochar to the soil can be used as an adaptation strategy by local farmers to cope with the effects of climate change in terms of adaptation to drought and water economy in irrigation. Nevertheless, more research is required to better understand the relation between biochar and soil moisture content at permanent wilting points and field capacity.

## Figures and Tables

**Table 1:** Basic soil and biochar composition.

Attribute	Soil	Biochar	Method of measurement
Soil texture	Sandy loam	—	Textural triangle [32]
pH	6.65	—	Potentiometric method
CE 1/10 (*μ*s/cm)	59.34	—	Conductivity cell
Carbon (%)	0.478	83.49	Oxidation (soil)/[33] (biochar)
OM (%)	0.824	—	Spectrophotometry
Nitrogen (%)	0.041	0.78	Kjeldahl (soil)/[33] (biochar)
Oxygen (%)	—	5.54	[33]
Hydrogen (%)	—	2.44	[33]
Calcium (meq/100 g)	6.447	—	Atomic absorption spectrophotometry
Magnesium (meq/100 g)	1.036	—	Atomic absorption spectrophotometry
Sodium (meq/100 g)	0.152	—	Atomic absorption spectrophotometry
Potassium (meq/100 g)	0.008	—	Atomic absorption spectrophotometry
Total phosphorus (ppm)	77.417	—	Bray and Kurtz [34]
Sulfur (meq/100 g)	7.633	—	Titrimetric method
Cation exchange capacity (meq/100 g)	12.225	—	Agronomic soil tests
Moisture content (%)	—	8.5	NF EN 1860-2
Volatile matter (%)	—	9.75	NF EN 1860-2
Ash content (%)	—	7.75	NF EN 1860-2
Biochar particle size (=0.05 mm)	—	6.5	Sieving method
Biochar particle size (=0.2 mm)	—	19	Sieving method
Biochar particle size (=2 mm)	—	46.5	Sieving method
Biochar particle size (>2 mm)	—	28	Sieving method

**Table 2:** Different levels of water and biochar and their combinations.

Water level	Biochar amount	Treatment label (B)	Treatment description
W0	B0	W00	Full irrigation-0 t·ha^−1^ biochar
W0	B1	W01	Full irrigation-15 t·ha^−1^ of biochar
W0	B2	W02	Full irrigation-30 t·ha^−1^ of biochar
W1	B0	W10	75% of full irrigation-0 t·ha^−1^ biochar
W1	B1	W11	75% of full irrigation-15 t·ha^−1^ biochar
W1	B2	W12	75% of full irrigation-30 t·ha^−1^ biochar
W2	B0	W20	50% of full irrigation-0 t·ha^−1^ biochar
W2	B1	W21	50% of full irrigation-15 t·ha^−1^ biochar
W2	B2	W22	50% of full irrigation-30 t·ha^−1^ biochar

Here, W0 = 8 L·m^−2^·day^−1^ (full irrigation); W1 = 6 L·m^−2^·day^−1^ (75% of full irrigation); W2 = 4 L·m^−2^·day^−1^ (50% of full irrigation) and B0 (control) = 0 t·ha^−1^; B1 = 15 t·ha^−1^; B2 = 30 t·ha^−1^.

## Data Availability

Data are available upon request from the corresponding author.
